# Chromosome Painting in *Gymnotus carapo* “Catalão” (Gymnotiformes, Teleostei): Dynamics of Chromosomal Rearrangements in Cryptic Species

**DOI:** 10.3389/fgene.2022.832495

**Published:** 2022-03-24

**Authors:** Milla de Andrade Machado, Maelin da Silva, Eliana Feldberg, Patricia Caroline Mary O’Brien, Malcolm Andrew Ferguson-Smith, Julio Cesar Pieczarka, Cleusa Yoshiko Nagamachi

**Affiliations:** ^1^ Laboratório de Citogenética, Centro de Estudos Avançados da Biodiversidade, Instituto de Ciências Biológicas, Universidade Federal Do Pará (UFPA), Belém, Brazil; ^2^ Departamento de Biologia Estrutural, Molecular e Genética, Universidade Estadual de Ponta Grossa, Ponta Grossa, Brazil; ^3^ Laboratório de Genética Animal, Coordenação de Biodiversidade, Instituto Nacional de Pesquisas da Amazônia, Manaus, Brazil; ^4^ Department of Veterinary Medicine, Cambridge Resource Centre for Comparative Genomics, University of Cambridge, Cambridge, United Kingdom

**Keywords:** amazon, banded knife-fish, whole chromosome painting, FISH, complex of species

## Abstract

The genus *Gymnotus* is a large monophyletic group of freshwater weakly-electric fishes, with wide distribution in Central and South America. It has 46 valid species divided into six subgenera (*Gymnotus*, *Tijax*, *Tigre*, *Lamontianus*, *Tigrinus* and *Pantherus*) with large chromosome plasticity and diploid numbers (2n) ranging from 34 to 54. Within this rich diversity, there is controversy about whether *Gymnotus* (*Gymnotus*) *carapo* species is a single widespread species or a complex of cryptic species. Cytogenetic studies show different diploid numbers for *G. carapo* species, ranging from 40 to 54 chromosomes with varied karyotypes found even between populations sharing the same 2n. Whole chromosome painting has been used in studies on fish species and recently has been used for tracking the chromosomal evolution of *Gymnotus* and assisting in its cytotaxonomy. Comparative genomic mapping using chromosome painting has shown more complex rearrangements in *Gymnotus carapo* than shown in previous studies by classical cytogenetics. These studies demonstrate that multiple chromosome pairs are involved in its chromosomal reorganization*,* suggesting the presence of a complex of cryptic species due to a post zygotic barrier. In the present study, metaphase chromosomes of *G. carapo occidentalis* “catalão” (GCC, 2n = 40, 30m/sm+10st/a) from the Catalão Lake, Amazonas, Brazil, were hybridized with whole chromosome probes derived from the chromosomes of *G. carapo* (GCA, 2n = 42, 30m/sm+12st/a). The results reveal chromosome rearrangements and a high number of repetitive DNA sites. Of the 12 pairs of *G. carapo* chromosomes that could be individually identified (GCA 1–3, 6, 7, 9, 14, 16 and 18–21), 8 pairs (GCA 1, 2, 6, 7, 9, 14, 20, 21) had homeology conserved in GCC. Of the GCA pairs that are grouped (GCA [4, 8], [5, 17], [10, 11] and [12, 13, 15]), most kept the number of signals in GCC (GCA [5, 17], [10, 11] and [12, 13, 15]). The remaining chromosomes are rearranged in the GCC karyotype. Analysis of both populations of the *G. carapo* cytotypes shows extensive karyotype reorganization. Along with previous studies, this suggests that the different cytotypes analyzed here may represent different species and supports the hypothesis that *G. carapo* is not a single widespread species, but a group of cryptic species.

## Introduction

The genus *Gymnotus* (Gymnotiformes, Teleostei) is a large monophyletic group of weakly-electric freshwater fish. It is the most speciose genus of the order, with 46 validated species (Craig et al., 2019; Kim et al., 2020; Fricke et al., 2021), widely distributed in the Neotropical region (Central and South America). The highest diversity is found in the Amazon-Orinoco-Guiana basins (Mago-Leccia, 1994; Albert, 2001; Albert and Crampton, 2005).

*Gymnotus* is divided into six subgenera: *Gymnotus*, *Tijax*, *Tigre*, *Lamontianus*, *Tigrinus* and *Pantherus* (Craig et al., 2019) with substantial chromosome plasticity. The diploid number (2n) varies from 2n = 34 in *Gymnotus capanema* (Milhomem et al., 2012a) to 2n = 54 in *G. carapo* (Foresti et al., 1984), *G. mamiraua* (Milhomem et al., 2007), *G. paraguensis* (Margarido et al., 2007) and *G. inaequilabiatus* (Scacchetti et al., 2011). The growing number of studies based not only on the karyotypic formula but also on different kinds of repetitive DNA sequences such as rDNAs, satellites, microsatellites and transposable elements (Milhomem et al., 2007; Claro, 2008; Milhomem et al., 2008; Scacchetti et al., 2011; Milhomem et al., 2012a; Milhomem et al., 2012b; da Silva et al., 2014; Utsunomia et al., 2014; Almeida et al., 2015; da Silva, 2015; da Silva et al., 2016; Machado et al., 2017; Utsunomia et al., 2018), have shown many different species-specific karyotypes and even population variants.

This large variation in 2n, however, is mostly found in the *G. carapo* subgenus, the previous *Gymnotus carapo* clade (Craig et al., 2019). In all the other subgenera, the diploid number varies from 48 to 54 chromosomes, many sharing the 2n = 52 that is found in the basal subgenus, the species *G. (pantherus) pantherinus* (Scacchetti et al., 2011; da Silva et al., 2011; da Silva et al., 2014; Utsunomia et al., 2014; Almeida et al., 2015; da Silva et al., 2016; Machado et al., 2017; da Silva et al., 2019), and also found in *Electrophorus*, the sister genus of *Gymnotus* (Fonteles et al., 2008; Cardoso et al., 2015).

In the subgenus *Gymnotus*, the *G. carapo* species has huge karyotype diversity among populations, with 2n ranging from 40 to 54, and many karyotypic formulas within the 2n described (Table 1). The species *G. carapo*, based on morphology and distribution data, was divided into subspecies *Gymnotus c. australis, Gymnotus c. caatingaensis, Gymnotus c. carapo, Gymnotus c. madeirensis, Gymnotus c. occidentalis, Gymnotus c. orientalis,* and *Gymnotus c. septentrionalis* (Craig et al., 2017). While most Neotropical fish species of South America have restricted geographic distributions, these species are distributed widely (Albert and Reis, 2011; Lehmberg, 2015; Craig et al., 2017).

**TABLE 1:** Cytogenetic studies of *Gymnotus arapaima, G. capanema and G. carapo*.

Species	2n (KF)	Authors	Localidades
*G. arapaima*	44 (24m/sm+20st/a)	Milhomem et al. (2013)	1. Reserva de Desenvolvimento Sustentável Mamirauá, AM
*G. capanema*	34 (20m/sm+14st/a)	Milhomem et al. (2012b), Milhomem et al. (2012a))	2. Capanema, PA
*G. carapo*	54 (54m/sm)	Foresti et al. (1984)	3. Miracatu, SP; 4. Botucatu, SP
	52 (50m/sm+2st/a)		5. Brotas, SP
	48 (34m/sm+14st/a)		6. Humaitá, AM
	42 (32m/sm+10st/a)		7. Belem, PA
	54 (52m/sm+2st/a)	Fernandes-Matioli et al. (1998)	8. Rio Mogi-Guaçu, SP
	54	Claro (2008)	9. Santa Albertina, SP;
			10. Cardoso, SP;
			11. Terra Roxa, SP;
			12. Mariápolis, SP
			13. Corumbataí, SP;
			4. Botucatu, SP;
			14. Angatuba, SP;
			15. Indaiatuba, SP
			16. São Lorenço, SP;
			17. Bertioga, SP;
			18. Piquete, SP;
			19. Cruzeiro, SP.
	42 (30m/sm+12st/a)	Milhomem et al. (2007)	20. Santa Cruz do Arari, PA;
	42 (30m/sm+12st/a)	Milhomem et al. (2008)	21. Ponta de Pedras, PA;
			22. São Miguel do Guamá, PA;
			2. Capanema, PA;
			23. Benfica, PA
	40 (28m/sm+12st/a)	Milhomem et al. (2008)	24. Almeirim, PA
*G.* cf. *carapo*	54 (50m/sm+4st/a)	Scacchetti et al. (2011)	25. Passo do Lontra, MS
*G. carapo* “Catalão”	40 (30m/sm+10st)	da Silva et al. (2014)	26. Lago Catalão, AM
*G. carapo* “Maranhão”	42 (30m/sm+12st/a)	da Silva et al. (2019)	27. Rio Munin, MA

Chromosome painting is as an important tool in comparative cytogenetics studies of fish species. The results have helped to solve various issues in this field and have giving insights into several evolutionary issues (Barby et al., 2019). It has helped to bring insights into the origin of B chromosomes (Vicari et al., 2011; Scudeler et al., 2015; Utsunomia et al., 2016), the origin and evolution of sex chromosomes (Carvalho et al., 2017; Cioffi et al., 2017; de Oliveira et al., 2017; de Moraes et al., 2017; Yano et al., 2017; de Freitas et al., 2018) and helped in the understanding of chromosomal evolution and relationships between closely related species (Ráb et al., 2008; Nagamachi et al., 2010; Nagamachi et al., 2013; Milhomem et al., 2013; Machado et al., 2018; Cioffi et al., 2019). Nagamachi et al. (2010) produced whole chromosome probes (WCP) from *G. c. orientalis* (GCA42, 2n = 42, 30m/sm+12st/a) by flow sorting, and hybridized these probes to the cytotypes of *G. c. orientalis* with 2n = 40 (GCA40, 34m/sm+6st/a). Two other studies with WCP from GCA42 mapped the karyotypes of *G. capanema* (GCP 2n = 34, Nagamachi et al., 2013) and *G. arapaima* (GAR 2n = 44, Machado et al., 2018). Those studies showed a higher level of chromosomal rearrangement than previously thought between these species.

In this study we used GCA42 WCP (Nagamachi et al., 2010) for mapping the karyotype of *G. c. occidentalis* “Catalão” (GCC 2n = 40), a distinctive population which has been proposed as a new species (da Silva et al., 2014). The results were compared with those obtained from GCA40 (Nagamachi et al., 2010), GCP34 (Nagamachi et al., 2013) and GAR44 (Machado et al., 2018).

## Material and Methods

### Sampling

**FIGURE 1:**
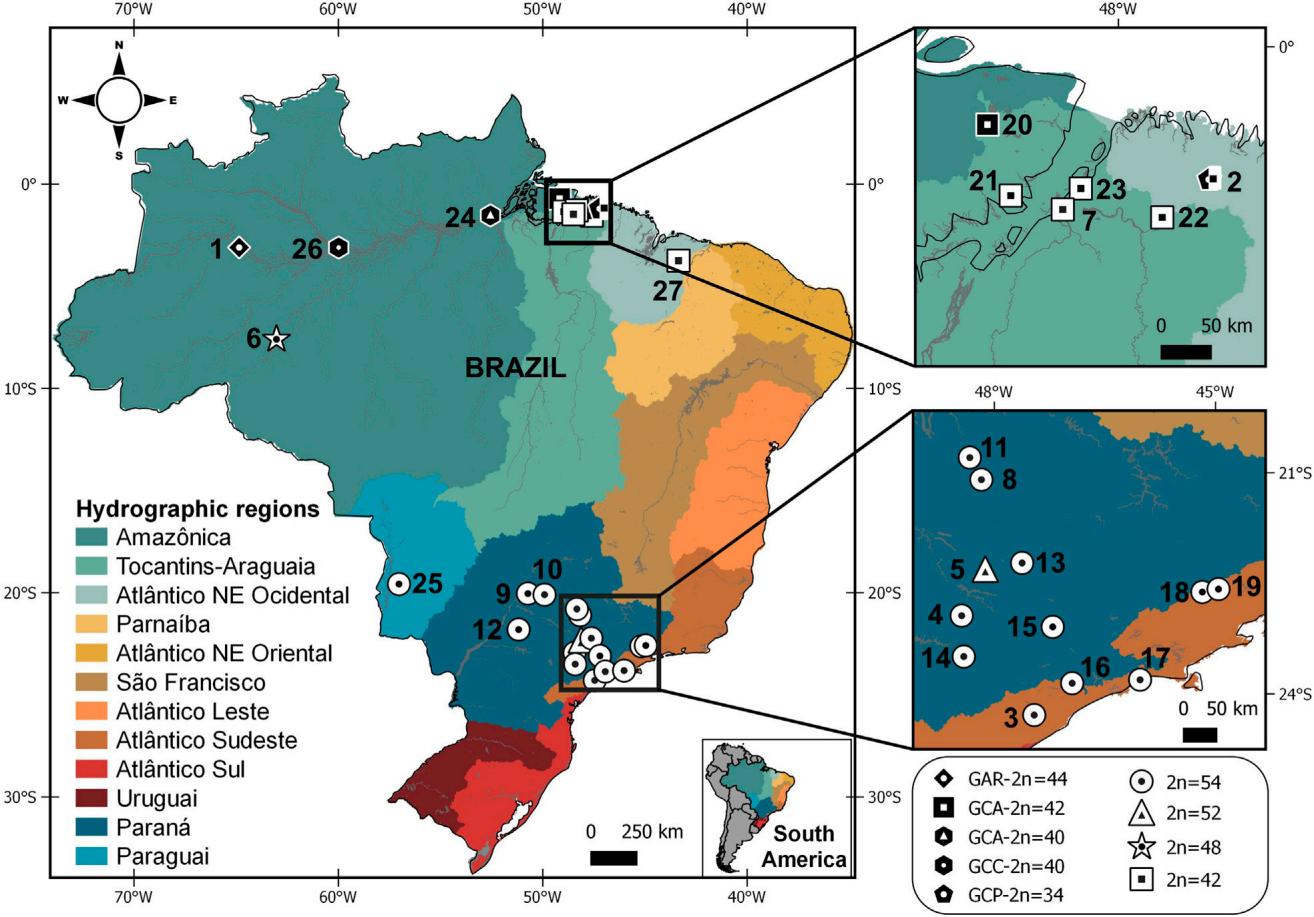
Map showing the Brazilian hydrographic regions and sample points for *Gymnotus arapaima*, *G. capanema*, and *G. carapo*, with karyotypic information (2n = diploid number) from the present study and from the literature. Each symbol indicates the karyotype obtained from the locality; more than one symbol indicates that distinct karyotypes were collected at the same locality. Symbols in white denote specimens analyzed by classic cytogenetics; symbols in black denote specimens analyzed by chromosome painting with *G.* carapo (2n = 42) whole-chromosome probes (Table 1; Nagamachi et al., 2010; Nagamachi et al., 2013; Machado et al., 2018, and present study). The numbers refer to localities detailed on Table 1.

Samples of *G. carapo* “Catalão” (GCC) were collected in Amazonas, Brazil (Figure 1). The Cytogenetics Laboratory from Centro de Estudos Avançados da Biodiversidade (UFPA) has permit number 19/2003 from the Ministry of Environment for sample transport and permit 52/2003 for using the samples for research. The Ethics Committee from Para Federal University (Comitê de Ética Animal da Universidade Federal do Pará) approved this research (Permit 68/2015). Sample collections were authorized by Instituto Chico Mendes de Conservação da Biodiversidade (ICMBio) and Secretaria de Estado de Meio Ambiente do Pará (SEMA-PA) under permit 020/2005 (Registration: 207419).

### Map

A distribution map was made using QGIS v.3.10.7. The shapefiles containing country limits were obtained from DIVA-GIS (Hijmans et al., 2004). We used the hydrographic regions limits provided by Braga et al. (2008) and we created the shapefiles on QGIS v.3.10.7. The localities numbered are shown on Table 1.

### Whole Chromosome Painting

Whole Chromosome Probes (WCP) from *G. carapo* (2n = 42; 30m/sm+12st/a; Nagamachi et al., 2010) were hybridized onto metaphases of *G. carapo* “Catalão”. Chromosome painting techniques followed Yang et al. (1995) with the modifications proposed by Nagamachi et al. (2010). Chromosomes were classified morphologically according to Levan et al. (1964) with modifications. The karyotype was organized following da Silva et al. (2014) *.*

## Results

### Chromosome Painting in *Gymnotus carapo Occidentalis* “Catalão”

**FIGURE 2:**
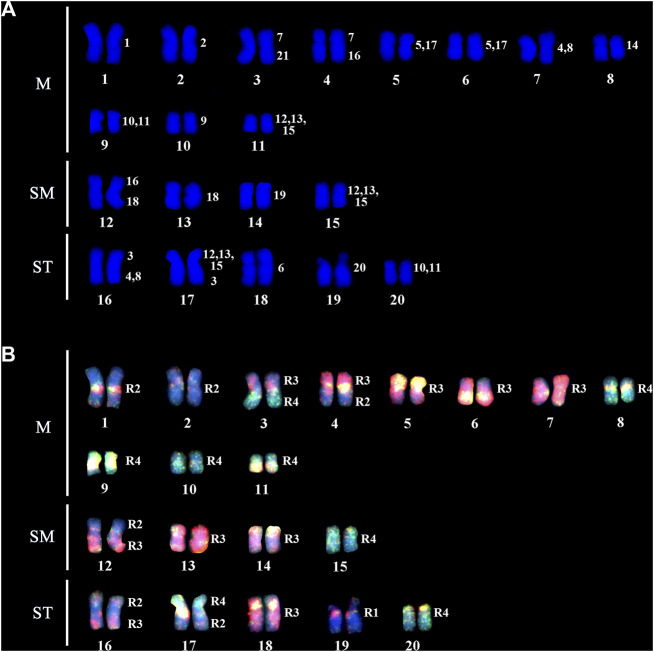
**(A)** A DAPI stained karyotype of GCC; the numbers on the right represent the *G.* carapo (GCA42) equivalent chromosomes. **(B)** Dual color fish with the probes of R3 (pairs 4–8 and 17–19; red) and R4 (pairs 9–15 and 21; green). Chromosome segments hybridizing with 2 colors indicate repetitive DNA sequences. The chromosomes or segments in blue (DAPI) represent the NOR-bearing chromosome of GCA42 (pair 20) and the chromosomes corresponding to R2 of GCA42 (pairs 1–3 and 16).

*Gymnotus carapo occidentalis* “Catalão” (GCC) has 2n = 40 with 30m/sm+10st/a chromosomes (Figure 2A) without differentiated sex chromosomes in male and female specimens.

The regions of homology with GCA42 are indicated on the karyotype of GCC arranged from DAPI-stained chromosomes (Figure 2A).

Dual color FISH with the probes of GCA42 from R3 (pairs 4–8 and 17–19; red) and R4 (pairs 9–15 and 21; green) define the chromosome groups in GCC40 corresponding to the four groups in Figure 2B. Chromosome segments hybridizing with 2 colors indicate repetitive DNA sequences. The chromosomes or segments in blue (DAPI) represent the GCA42 NOR-bearing chromosome (pair 20) and the chromosomes corresponding to R2 (pairs 1–3 and 16).

From the 12 chromosome pairs of GCA42 that can be individually differentiated (pairs 1–3, 6, 7, 9, 14, 16 and 18–21), 8 pairs (1, 2, 6, 9, 14, 19, 20, 21) conserve homeology within GCC40 (pairs 1, 2, 3, 8, 10, 14, 18, 19). GCA42 pair 20 hybridizes one whole chromosome in GCC40, pair 19. Four chromosome pairs of GCA42 (3, 7, 16, and 18) show 2 signals on GCC40 (Figure 2).

The GCA42 probes that represent two chromosome pairs (4,8), (10,11) and (5,17) all reveal 2 signals, and the one that represents three pairs (12, 13, 15) reveals 3 signals on the GCC40 chromosomes.

The following chromosome associations of GCA42 are present in GCC40 pairs: 3 (7/C/21); 4 (7/C/16); 12 (16/C/16/18); 16 (3/C/3/[4, 8]); 17 ([12, 13, 15]/C/[12, 13, 15]/3), where C = centromere.

### Comparative Chromosome Painting Analysis Among Different *Gymnotus* Karyotypes

**FIGURE 3:**
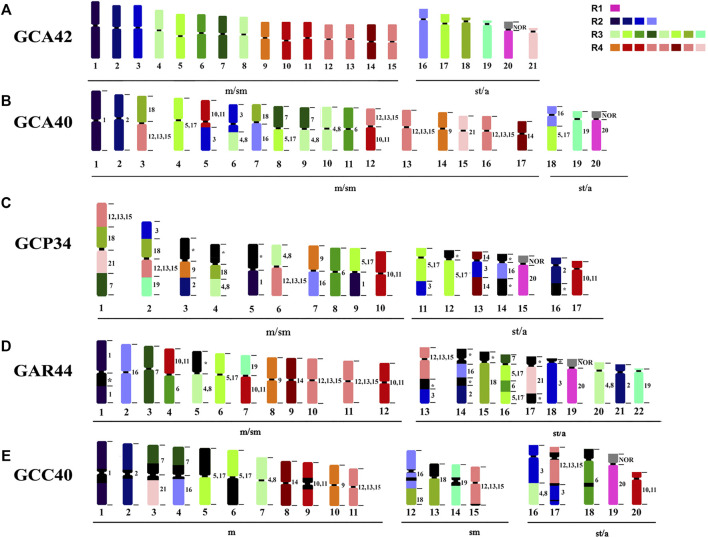
Ideogram showing chromosome rearrangements obtained by WCP. R1, R2, R3 and R4 representing the colors of the four major regions of the *G. carapo* 2n = 42 probes. **(A)** *G. carapo* 2n = 42 (GCA42) utilized in the production of the probes, each different color represents a chromosome or chromosome group; **(B)** *G. carapo* 2n = 40 (GCA40) (Nagamachi et al., 2010); **(C)** *G. capanema* 2n = 34 (GCP34) (Nagamachi et al., 2013); **(D)** *G. arapaima* 2n = 44 (GAR44) (Machado et al., 2018); **(E)** *G. carapo* “catalão” 2n = 40 (GCC40) (present study).

**FIGURE 4:**
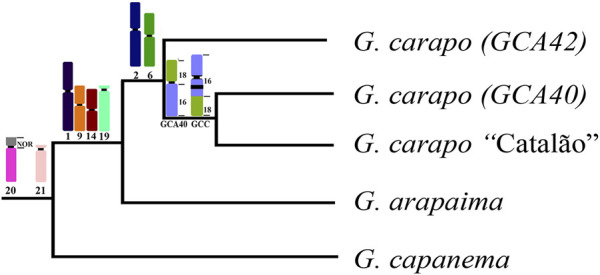
Representative phylogeny based on da Silva et al. (2019), with the syntenic blocks shared by the nodes. Chromosome numbers refer to the *G. carapo* 2n = 42 chromosomes (see Figure 3).

From the 12 chromosome pairs in GCA42 that can be identified individually (1–3, 6, 7, 9, 14, 16, 18–21), GCC shows conserved synteny in 8 pairs (1, 2, 6, 9, 14, 19, 20, 21); GCC shares the same 8 pairs with GCA40, and also the same eight pairs shared between GCA40 and GCA42, grouping them together and closer to each other than the other analyzed karyotypes. From the pairs that are grouped in GCA42 [(4, 8), (10,11), (5,17), (12, 13, 15)], all keep the same number of signals in GCC40, while GCA40 has an extra signal for (5,17) and for (12, 14, 15) (Figure 3). The 16/18 syntenic associations are shared between GCC40 (16/C/16/18) and GCA40 (18/C/16) indicating pericentric inversions (Figures 3, 4).

Compared to GAR, GCC shares five of the individually identified chromosomes (1, 9, 14, 20, 21), as well as the same number of signals in the groups (4, 8) and (12, 13, 15). There is also a similar chromosome rearrangement in GCC 17 and GAR 13 that is not shared with the other *G. carapo* cytotypes. However, as it is not possible to differentiate between GCA (2n = 42) 12,13, and 15, we cannot infer that the involved chromosome is the same or is different (Figure 3; Table 2).

**TABLE 2:** Syntenic blocks shared among analyzed species with WCP. GCA42—*Gymnotus carapo* 2n = 42; GCA40—*G. carapo* 2n = 40; GCC40—*G. carapo* “Catalão” 2n = 40; GAR44 - *G. arapaima* 2n = 44; GCP34—*G. capanema* 2n = 34.

Species	Syntenic blocks
GCA42 x GCA40	GCA42 1, 2, 6, 9, 14, 19, 20, 21
GCC40 X GCA42	GCA42 1, 2, 6, 9, 14, 19, 20, 21
GCC40 X GCA40	GCA42 1, 2, 6, 9, 14, 19, 20, 21
GCC40 X GCA40 X GCA42	GCA42 1, 2, 6, 9, 14, 19, 20, 21
GCA42 x GAR44	GCA42 1, 9, 14, 18, 20, 21
GCA40 x GAR44	GCA42 1, 9, 14, 20, 21
GCC40 X GAR44	GCA42 1, 9, 14, 20, 21
GCC40 X GCA42 X GCA40 x GAR44	GCA42 1, 9, 14, 20, 21
GCA42 x GCP34	GCA42 6, 19, 20, 21
GCA40 x GCP34	GCA42 6, 19, 20, 21
GCC40 x GCP34	GCA42 6, 19, 20, 21
GAR44 x GCP34	GCA42 20, 21
ALL	GCA42 20, 21

Compared to GCP, GCC shares three individual pairs (GCA 1,20,21) and the same number of signals as GCA (4,8), (10,11) and (12, 13, 15). All species share homeology to GCA 1,20,21 (Figure 3; Table 2).

The syntenic block of GCA42 6 is conserved in four of the five analyzed karyotypes by painting, except for GAR (Figure 3; Table 2), in which it is divided into two signals in pairs 4 and 16, while the syntenic block 18 of GCA 42 is shared with GAR, but not with GCC or GCA 40 (Figure 3; Table 2).

## Discussion

*Gymnotus carapo occidentalis “*Catalão*”* has 2n = 40 (GCC, 30m/sm+10st/a), the same as *G. carapo orientalis* (GCA) 2n = 40 (28m/sm+12st/a), but with a different karyotype. It is hypothesized that the basal diploid number for Gymnotidae is 2n = 52 (da Silva et al., 2019), as the sister species *Electrophorus electricus* and *G. pantherinus* (sister species to all *Gymnotus*, Craig et al., 2019) both have 2n = 52.

This variation in karyotype can be consistently observed along the hydrographic regions. The 2n = 42 is found in the *G. c. orientalis* locations in the “Tocantins-Araguaia” region, while the 2n = 40 is observed in the *G. c. orientalis* (GCA40) located in the “Amazonica” region. While sharing the same 2n GCA40, GCC was sampled in a lake in an area close Negro river, in *G. c. occidentalis* occurrence area and also has significant karyotype differences. The 2n = 48 is found only in *G. c. madeirensis* (Table 1; Figure 1). The higher 2n = 52 and 54 is found only in *G. c. australis*, distributed along the Paraguai, “Paraná” and “Atlântico Sudeste” hydrographic regions.

When compared to all cytotypes of *G. carapo* in the literature, there is a tendency to a reduction in diploid number in the “Amazonica”, “Tocantins-Araguaia” and “Atlântico Nordeste Ocidental” hydrographic regions, a trait also shared with *G. arapaima* (Figure 1). Whereas the cytotypes in the “Paraguai”, “Paraná” and “Atlântico Sudeste” hydrographic regions have a higher chromosome number, with all locations having 2n = 54 except in one population that has 2n = 52 (Table 1; Figure 1) similar to more basal 2n in the genus. This suggests that the reduction in diploid number in the amazon region happened after colonization of the area.

Whole chromosome probes of GCA42 have been used in previous studies comparing two cytotypes of *G. carapo* (GCA42 and GCA40), *G. capanema* (GCP34) and *G. arapaima* (GAR44). The results demonstrate highly rearranged karyotypes, more than found by classical cytogenetics alone (Nagamachi et al., 2010; Nagamachi et al., 2013; Machado et al., 2018). The same results are observed in *G. carapo* “Catalão” (present study), confirming that the chromosomal evolution in this group is quite complex.

The karyotypes of the three *G. carapo* cytotypes analyzed by chromosome painting (GCA42, GCA40 and GCC40) are more similar to each other than to those of GAR44 or GCP34 and share a uniform amount of synteny. However, they have multiple species-specific rearrangements, which probably constitute a post zygotic barrier that would result in an inviable or infertile hybrid (Figure 4). We observe the same pattern when compared to *G. arapaima*, explained by the fact that they are sister species with recent divergence (Brochu, 2011) in relation to *G. capanema* (Craig et al., 2019).

This large number of chromosomal rearrangements in *Gymnotus carapo*, demonstrated by chromosome painting, indicates that the different cytotypes constitute a complex of cryptic species as already suggested (Milhomem et al., 2008; Nagamachi et al., 2010). The chromosomal speciation must have played a key role in this process that, if associated with small-inbred demes, could have facilitated the fixation of chromosomal rearrangements (King, 1993).

Currently these cryptic species (with the same morphology but different cytotypes) are in allopatry, which corroborates the pattern found in many Neotropical freshwater fish groups. This could be due to the dynamics of the river networks, including the fragmentation and merging of adjacent rivers, that led to increased species richness closer to the core region of the Amazon basin (Albert and Reis, 2011; Tagliacollo et al., 2016; Albert et al., 2018a; Albert et al., 2018b; Albert et al., 2020).

The taxonomy of *G. carapo* is quite complex and has been discussed in many studies. Some authors report it as a single generalized species (Craig et al., 2017), other studies show a paraphyletic group within a monophyletic complex of related species (Brochu, 2011; da Silva et al., 2019) and still others suggest it to be a complex of cryptic species (Milhomem et al., 2008; Nagamachi et al., 2010). Within this context, some phylogenetic studies with molecular data show polytomy of the *G. carapo* complex, consisting of *G. carapo*, *G. arapaima*, and *G. ucamara* (Lovejoy et al., 2010; Brochu, 2011) and other studies show species of the subgenus *Gymnotus* nested within *G. carapo* lineages (Lehmberg et al., 2018; Craig et al., 2019; da Silva et al., 2019).

In conclusion, the results presented here support that these populations with different cytotypes of *G. carapo* analysed (*G. carapo occidentalis “Catalão”*, *G. c. orientalis* GCA42 and GCA 40, along with the geographic-specific 2n = 48 and 2n = 54) may be a cryptic species complex. Analyses by chromosome painting of more cytotypes of *G. carapo* as well as other species of this genus coupled with molecular studies of those samples could help elucidate the chromosomal evolution and pattern of speciation in the group and help identify same-species populations from endemic species that have recently diverged.

## Data Availability

All data presented in this study are found in the article.
